# Oral Surgery: Theory and Results

**Published:** 1897-06

**Authors:** G. Lenox Curtis


ARTICLE VII.

### ORAL SURGERY: THEORY AND RESULTS.

BY G. LENOX CURTS, M. D.

Read before Canadian Medical Association.

Theories in surgery, as in finance or government, when founded on insufficient data, are apt to be exploded when an attempt is made to demonstrate their real worth by practical test. For it is undeniable that results are the true criteria of the value of work. A theory which will not at all times bear this test falls.

With general surgery and many of its special branches have been brought to a high degree of perfection, where theory and practice accord beautifully, there are departments of the great work of no less importance than the fields, now cultivated by the medical profession, which are utterly neglected in the teachings of the medical institutions and in the practice of medical men. The physician considers it beneath his dignity to investigate the mouth as an indicator or cause of disease further than to look at the tongue. He will not refer to the teeth lest he may be classed with the "dentists." Yet the mouth, which is the gateway to the alimentary tract, the portal through which passes the food which nourishes the body, would seem to demand his first and closest consideration.

The completeness of the lack of knowledge on the part of the average physician and surgeon, concerning diseases attendant upon or following affections of the teeth, of the effects, near and remote, which such affections may cause in the organism, is appalling. Many times their patients suffer untold agony or endure prolonged illness because of the doctor's ignorance upon these subjects, which should be among the fundamentals. For which, if not all, of this the medical institutions of learning are responsible. In the curricula of many of these the teeth, in spite of all the attention that is given to them and their diseases, let alone their anatomical and nervous relations to the remainder of the economy, might well be foreign bodies. In view of all this, it may not be an unprofitable investment of the time to devote twenty minutes to the discussion of a few reports from a plain record of facts.

Mr. A., aged twenty-eight years (who, up to July 1st, 1894, had been in good health, well nourished, and above the average in physical development), in the latter part of March experienced trouble in the eruption of the right inferior wisdom-tooth.

Examination by his dentist revealed inflammation of the gums surrounding an impacted wisdom-tooth, but not sufficiently developed to lead him to do anything for relief of patient. The first and second molars were in position. Dismissed with advice if trouble continued to call again. Soreness increased until May, when the gum over the tooth was lanced and painted with tincture of iodine. This was several times repeated until June 1st, when an abscess was formed. This also was lanced and treated in like manner until July 1st, when the suffering of the patient caused the dentist to advise the extraction of the tooth, and the address of a professional extractor, whose knowledge of surgery was evinently based on theory, was given. Under nitrous oxide an attempt was made to extract the tooth, which resulted in the removal of the alveolar process on the lingual surface. When the patient recovered consciousness he was assured that the tooth had been fully extracted, and was shown a piece of bone of considerable size that had been taken out. He experienced excessive pain and discomfort from the operation, and there was great soreness, due to the lacerated tissue. Complaining of this, the extractor said it was of no importance, he would be all right in a day or two, not even prescribing a disinfectant mouth wash. Patient noticed considerable excitement on the part of the dentist and his assistant, and recalled hearing, while in a semi-conscious condition, the associate express surprise, which led him to believe that the operation was out of the usual run. For several days the patient was confined to his room, and unable to lie down because of the severe soreness of a bruised back. His jaws became rigid and closed, necessitating the use of a fluid diet. Face was badly swollen and pain increased hourly. On the second day following the operation pus began to flow from the mouth, and the swelling was so pronounced that he again consulted the extractor, who laid the trouble to cold and malaria, and considered the operation a perfect success.

After a week of intense suffering and extreme weakness for want of food, he consulted his family physician, who was unable to relieve his suffering, and advised him to see a general surgeon, who in turn told him he was a subject for the dentist, and that he knew nothing of such diseases. Another surgeon, placing his finger along the inside of the cheek back to the upper wisdom-tooth, which had been fractured during the attempt to extract the wisdom-tooth, said that the whole trouble was there, and again he was referred to the dentist, who ridiculed and dismissed him as before. There was excruciating pain in the region of the right tonsil, which was relieved on opening of abscess. A physician was then called, who admitted his inability to treat the case, and turned him over to a young man with a recent hospital experience, in whose hands he got his first relief.

Pus had burrowed through and formed a large cheek abscess. The patient was now very weak and debilitated, with constant discharge of pus from the mouth. An opening was made through the face in the region of the malar bone, which was syringed daily. Under this treatment the patient improved and the swelling subsided, leaving an opening below and back of the angle of the inferior maxillæ, which was caused by the pus. Through the poison in his system the patient was in so precarious a condition that a consultation was held, and he was advised to go to New York for treatment. With his physician he appeared in my office on December 7th.

Examination revealed a large indurated mass just below the jaw on the right side. Very offensive pus was discharging from the opening before referred to. The introduction of a probe revealed extensive destruction of tissue below the jaw and extending back to the tonsil, where a hardened substance about an inch in size was outlined. Had not the history of the extraction been so definite, I would have been led to believe that a tooth was lodged there; but we concluded it was a fragment of the alveolar process covered with fibrous tissue, which his physician had been trying to dissolve with medicine given internally. The patient was able to separate his teeth one quarter of an inch, which allowed me to examine the wound where the tooth had been extracted. Pus was exuding freely from this wound of the same offensive character that predominoted. Passing a probe into the wound I found that the alveolar process had been fractured, and that a large opening led to the hardened mass external to the tonsil. The patient was in constant pain, confined largely to the right side of the face, very anæmic and nervous, and health completely broken. An immediate operation was advised. Under an anæsthetic the wound where the tooth had been was enlarged, rough bone due to the fracture of alveolar process and suppurating tissue leading to the hardened mass was curetted, allowing more complete examination for the cause of the trouble, when a steel probe readily detected enamel. I passed an instrument around and behind the tonsil, and gradually dislodged and removed a wisdom-tooth, upon sight of which his physician said, "It would take me a long time to dissolve that with any agent known to medicine."

The abscess under the jaw, which involved the entire cellular tissue was thoroughly curetted, leaving a depression about two by three inches in size with exceedingly thin skin. Packed with gauze and allowed the wound to granulate from bottom. No pus from wound near the tonsil after the operation, but some in outside wound until it was curetted.

Patient began at once to improve on antipyemic treatment and nourishing diet. Several days after the operation, he complained of severe pain extending along the right side of the face. Examination of the superior third molar revealed an exposed pulp, following the extraction of which there has been no return. In three weeks the patient was dismissed, and with no deformity from the operation and no return of trouble.

It seems reasonable to me that when the dentist attempted to extract the tooth, instead of grasping it he bore down upon its masticating surface with the point of the forceps, forcing the tooth through the alveolar process, which was fractured, and crowded the tooth behind the tonsil, where it was found embedded. I am told by the patient that since the extractor learned of the removal of the tooth he stated that he thought the patient had swallowed it, and did not dare to acknowledge the facts, preferring to cover up the wrong-doing by saying he had extracted the tooth and it had been lost among others in the cuspidor.

Mr. B., aged fifty-five years, suffered from neuralgia, which the dentist thought was due to abscess of the inferior left central incisor, and inferior left molar, which were pulpless. These teeth had been under treatment for some time, but resisted all efforts to be cured. On examination the upper arch was found edentulous, the patient wearing artificial teeth. Examination of diseased inferior incisor revealed a canal thoroughly opened, and a fistulous opening through the gum at the end of the root, through which a probe showed extensive absorption of the bone. The left lateral and cuspid teeth were found to contain decomposed pulps, and a probe could be passed from the fistula back to the bicuspid below the ends of the roots. The molars were also abscessed, with a fistulous opening through the gum on the lingual surface.

The posterior canal was opened through the apex, and the anterior buccal canal was partially entered and plugged with bamboo. Inferior wisdom tooth lost. On November 27th, 1894, the central incisor canals were cleansed, sterilized, and filled to the apex with chloropercha.

The canal in the lateral incisor was opened freely and drilled nearly to the apex, but I was unable to get nearer than a fraction of one-sixteenth of an inch from the apex. Sterilized and filled with chloropercha. Patient was referred to dentist for removal of gold crown from cuspid root, which was abscessed, and to report on Saturday. On that day examination revealed the removal of crown; the canal of the cuspid was more fully opened into and dressed with creosote.

On December 1st, canal of cuspid was more fully opened and a probe passed beyond the apex. Canals sterilized and filled with chloropercha, some of which oozed out through the apical foramen. Cocaine was injected into the gum and alveolotomy performed. Chloropercha oozed out through the wound. Cavity in alveolar process around cuspid and incisors burred and curetted. Debris washed out and wound sterilized. December 3rd, the gums over the cuspid found considerably swollen. Wound opened with probe and tincture of iodine injected. December 5th, gums found less swollen and less inflamed. External application of iodine. Anterior buccal canal of molar opened to apex; also posterior canal more freely opened to apex. Search for lingual canals, from which the abscess started, resulted more favorably after drilling considerable dentine away to the floor of the pulp-chamber. Canals found to be small and almost closed by deposit of secondary dentine, but larger upon opening into them. Both were opened to the apex, so that a delicate probe passed beyond. All four canals were flooded with carbolic acid; ropes of cotton were packed in and sealed for the purpose of disinfection. Two hours' time was occupied in opening these canals. The following day the canals were packed with iodoform. No unusual disturbance around tooth. December 7th, all signs of inflammation had subsided and the teeth were entirely comfortable. The canals were dried and filled with chloropercha, which was forced through the apical foramen of the distal canals, and oozed through the fistula in the gum. The floor of the pulp-chamber was carefully lined with gutta-percha and the cavity filled with cotton. Case referred to dentist for filling. Under cocaine alveolotomy was performed, abscess and debris burred and curetted; wound washed out with electrozone. Wound dressed daily for several days with disinfectant and tincture of iodine. Patient complained all the time of severe neuralgic pain in the left side of face, more especially when tired or at night. I directed the dentist's attention to second left inferior molar, which was very sensitive, owing to abrasion and its having been ground down so as to make the teeth on the plate above occlude properly. Advised to look for irritation of pulp. This advice was not considered good to the extent of investigation. December 19th, after an exceedingly restless and painful night, patient consulted family physician, who bitterly censured the advice and operations of the dentist and of myself, and demanded that he immediately go to a professional extractor and have the teeth drawn, leaving the posterior molar untouched. It was no easy task for me to dissuade the patient from acting on the physician's advice. Again I repeated the necessity of care of back molar; I also opened through the gum and curetted around the anterior buccal root of the first molar with a view to blood-letting and to relieve light congestion around tooth, and also in the pulp of back molar. The pain continued, and the dentist reluctantly saw the wisdom of opening into the second molar, which revealed four pulp-stones about the size of a pin's head as the cause of the trouble, on the removal of which, along with the entire pulp, all pain disappeared and the patient was rendered comfortable. Since this treatment patient has enjoyed best of health.

Mr. D., aged thirty-four. For ten years or more had dull pain in upper right half of face, sometimes extending to side of head with soreness in upper jaw before malar bone. About seven years prior he had the first superior molar extracted, since when he noticed an opening through the gum in the neighborhood of the affected tooth, through which pus discharged. All these years he at times had very heavy dull feeling in the right side of the face, in the nose, and under the eye, which would leave the eyeballs sore and tender. A pain sometimes ran down the right arm and side of the chest, resembling that of rheumatism. For one year he had continual sharp pain in the left side of the face and in the eye, which on any quick movement of head or an attempt to read rendered him dizzy so he would stagger. When apparently free from pain a quick turn of the head would cause it to reappear. Neither physician or dentist could point out the cause of the trouble, but advised the extraction of the second bicuspid on the affected side, where the pain centered, and an attempt to do so resulted in the fracture of the root, when, in order to get the pieces, nearly the entire alveolar process surrounding it was cut away. The wound was a long time in healing, and the neuralgic pains continued just the same. One of the surgeons whom he consulted gave him a placebo; from which I inferred, as did the patient, that the surgeon took his case to be one of hypochondriasis.

He appeared at my office with the history given above. Examination of the right side revealed a fistulous opening posterior to the second bicuspid, which would be exceedingly difficult for inexperienced eyes to detect, as there was no inflammation or hypertrophy surrounding it. A delicate probe was readily passed through it and into the antrum. The removal of the probe was followed by a straw-colored fluid, leading me to the belief that bone disease existed. I found that all the alveolar process anterior to the second molar roots and first bicuspid, save sufficient to hold that tooth in position, had been destroyed. The destruction extending to the lower border of the malar bone and floor of antrum. Within that area it was completely gone. The hard palate opposite the extracted tooth was necrosed for half an inch and a sequestrum about one quarter of an inch in width, held and supported by a narrow neck, was forming at the line of demarcation. The second bicuspid contained a putrescent pulp, which when opened was exceedingly offensive. I concluded cause sufficient for disturbance on the right side of the face had been detected. The symptoms on the left side were then looked into. The left half of the upper lip was swollen and inflamed, especially so at night, bothering him in talking and eating. A notable condition in the expression of that half of the face was the want of normal fullness showing a long-continued irritation of the nerves which supplied the muscles, which had resulted in their being atrophied, save those of the upper lip; even a change in the size and expression of the eye was visible. During the examination he had many paroxysms of pain. The inferior left wisdom-tooth had been extracted. The teeth in upper jaw excepting the first molar had a normal appearance. The wisdom tooth was elongated from lack of occlusion, and the gum around the same was slightly inflamed. Percussion of the teeth produced normal notes save in the first molar, in which the discord was very faintly heard, there was no soreness of tooth, but a change in the expression of the eye led to the belief of pulp-stones occupying it. There was also faint discoloration. The mesial buccal root was slightly denuded of gum tissue, due to extraction of the bicuspid and the iujury to the gum. The cementum of the denuded root was not sensitive to any of the usual tests made, and I decided to open through the side of this root with a small drill to ascertain its vitality. The canal was entered with no visible signs of a pulp. No odor present to indicate that it was dead. I passed a flexible bristle to the apex of the root without resistance. A drop of blood was drawn from beyond the apex. A large opening was then drilled through the masticating surface of the tooth, and the pulp chamber was fully exposed and found to contain three large pulp-stones. One covered the canal of the palatal root, and upon its removal the entire pulp of that root came away attached and completely ossified except a sixty-fourth of an inch at the apex. Its removal was followed by a gush of blood.

The cure was like magic. The patient's general impression changed instantly, as if he had been freed from captivity, and he exclaimed: "Why, what have you done? The pain has gone; I can turn my head quickly without causing pain." Believing this statement to be true, and to immediately make further test, I said, "Perhaps it is only mental relief," upon which he remarked, "Mental or not, there certainly is a change, and I am free from pain"; and this condition has proved to be lasting, as well as true. All three canals were opened to the apex, filled with wax, and left for the dentist to finish. The patient reported having had the first night of uninterrupted sleep in over a year, with no pain. He also had been able to read without vertigo, and he very much enjoyed the ability to move his head quickly without anticipation of suffering. Not finding indications of pulp-stone in the wisdom-tooth, and it being no practical value to the patient, because of the loss of the antagonizing tooth, I extracted it with the view of examining the pulp, and thus prove tests of diagnosing pulp stones, and was gratified on opening the tooth to find a normal pulp. This is contrary to theory by surgeons who claim that pulp-stones only appear in teeth that have lost their antagonist. Thorough cleansing, sterilizing, and filling of the canals of the bicuspid in the right side followed, Examination of the antrum did not reveal a purulent condition, only a chronic inflammation. The discharge of pus was from the necrosis of the palatal plate. The operation consisted of burring away the sequestrum, abscess sac, and granulating tissue, curetting and removing same, and douching both the alveolar cavity and antrum with peroxide and bichloride solutions, and repeating the bichloride twice daily for forty-eight hours. Ten days later the report from the patient was very favorable, no inconvenience whatever having been experienced. This is one of many cases with such satisfactory results.

Another typical case bearing on the subject in hand is reported to me by my friend, Dr. Ives.

A lad, twelve years of age, was brought to him November, 1894, for dental operation. Examination of the mouth showed an over-crowded arch, resulting in irregularity of the teeth, which were very poorly calcified, and contained many sensitive cavities. In the inferior first molars were extensive amalgam fillings and several disintegrating spots. The pulps of the superior first molars were dead, and in reply to an inquiry as to why the lad wore glasses, his mother said, "By order of his physician, under whose care he has been for a long while for treatment of 'St. Vitus' dance of the eyes.' " The boy's eyes, lids and brows were rapidly and constantly twitching, to the great discomfort of himself and those aboat him, and he was nervous and irritable. Dr. Ives's experience enabled him to quickly see the relation between the boy's trouble and the condition of his teeth, and he directed that he be taken to Dr. Hasbrouck for the extraction of the four sixth-year molars, with the assurance that the extraction would cure his "St. Vitus' dance." This was done, and at the expiration of ten days the boy returned, without glasses, and all signs of irregular movements about the eyes had disappeared. The boy was then taken to the physician, a well known oculist of good repute, with a statement of what had been done, but he repudiated the idea that the change was owing to the extraction of the teeth. "It was impossible," he said, and claimed that the cure was entirely due to his own treatment.

These cases point to the idea previously expressed of the lack of appreciation among members of the medical profession generally, of the important *role* which the condition of the mouth and teeth, more especially the latter, plays in disease. They can be duplicated by the dozen, but hundreds, alas, of the sufferers from the protean effects of unsuspected dental disease never find relief because of the ignorance of their physicians. There are many cases, again, when the dentist discovers the cause of the trouble with the patient's general health, but he is overruled by the physician, whose authority and knowledge are supposed by the patient to be supreme.

Few, perhaps, have better opportunities than I to see the evils which flow from the physician's ignorance upon the subject of the teeth. The physician, in formulating his theories for the explanation of obscure troubles, entirely ignores this factor. He has never been taught to appreciate the teeth as a possible element in any disorder, except toothache, or perhaps a neuralgia of the face. The medical schools are no aid to him, the text-books give no inkling of the teeth. The teeth are the province of the dentist, and the dentist is too often looked upon with contempt by his medical confrere as being a one-sided, semi-educated man, when really this very one-sidedness has made him a master in oral and facial diseases. Upon these points the dentist does not vainly theorize. He gets results, and these results are his recommendations to the medical profession.

It seems to me that in this day of enlightenment upon the teeth among dentists, it is almost criminal in the medical institutions of learning to send their graduates out with less than half the knowledge which should be given them. If this be so with regard to the college, what shall we say of the man, who precticing the most beneficient profession in the world, fails to acquaint himself with a subject so important to the sound pursuit of medicine? Is he not lacking in his duty to himself and to his patients? With so important a factor, in many cases entirely omitted, can he do more than vainly experiment upon his patients, blindly grasping for what he has not eyes to see?

I have no objection to experiment with patients, with a view to further enlightenment, provided it is done honestly and with all the possible known elements estimated at their true value. But when experiment is necessary because the physician or surgeon lacks common practical knowledge which he can easily avail himself of, I cannot uphold it. Such a course must necessarily be merely mercenary. A theory formed by such a man must be wrong, his practice cannot help being mischievous, his results, so far as good is concerned, will be nil. He is simply a "guesser," and while he is guessing his patient's life may be slipping away.

It behooves us, then, to endeavor by every manly means to free ourselves from every environing circumstances which tends to cramp our efforts to relieve human suffering. What we are after are results, not theory. As we can learn industry from the little busy bee or patience and perseverance from the spider, so we may even learn from the dentists the relations of the condition of the teeth to apparently unrelated lesions. Certainly we should neglect no source of information which would strengthen or enlarge our means of fighting disease. So, and so only, shall we be able to confer upon our patients the highest benefits within the limits of our profession.—*Dominion Dental Journal.*

